# Health care cost and benefits of artificial intelligence-assisted population-based glaucoma screening for the elderly in remote areas of China: a cost-offset analysis

**DOI:** 10.1186/s12889-021-11097-w

**Published:** 2021-06-04

**Authors:** Xuan Xiao, Long Xue, Lin Ye, Hongzheng Li, Yunzhen He

**Affiliations:** 1 Eye Center, Renmin Hospital of Wuhan University, Wuhan, 430060 China; 2 School of Public Health, Fudan University, Shanghai, 200433 China; 3 Department of Eye Plastic and Lacrimal Disease, Shenzhen Eye Hospital of Jinan University, Shenzhen, 518040 China

**Keywords:** Glaucoma screening, Health economics, Artificial intelligence (AI), Grassroots community health care

## Abstract

**Background:**

Population-based screening was essential for glaucoma management. Although various studies have investigated the cost-effectiveness of glaucoma screening, policymakers facing with uncontrollably growing total health expenses were deeply concerned about the potential financial consequences of glaucoma screening. This present study was aimed to explore the impact of glaucoma screening with artificial intelligence (AI) automated diagnosis from a budgetary standpoint in Changjiang county, China.

**Methods:**

A Markov model based on health care system’s perspective was adapted from previously published studies to predict disease progression and healthcare costs. A cohort of 19,395 individuals aged 65 and above were simulated over a 15-year timeframe. Fur illustrative purpose, we only considered primary angle-closure glaucoma (PACG) in this study. Prevalence, disease progression risks between stages, compliance rates were obtained from publish studies. We did a meta-analysis to estimate diagnostic performance of AI automated diagnosis system from fundus image. Screening costs were provided by the Changjiang screening programme, whereas treatment costs were derived from electronic medical records from two county hospitals. Main outcomes included the number of PACG patients and health care costs. Cost-offset analysis was employed to compare projected health outcomes and medical care costs under the screening with what they would have been without screening. One-way sensitivity analysis was conducted to quantify uncertainties around model results.

**Results:**

Among people aged 65 and above in Changjiang county, it was predicted that there were 1940 PACG patients under the AI-assisted screening scenario, compared with 2104 patients without screening in 15 years’ time. Specifically, the screening would reduce patients with primary angle closure suspect by 7.7%, primary angle closure by 8.8%, PACG by 16.7%, and visual blindness by 33.3%. Due to early diagnosis and treatment under the screening, healthcare costs surged dramatically to $107,761.4 dollar in the first year and then were constantly declining over time, while without screening costs grew from $14,759.8 in the second year until peaking at $17,900.9 in the 9th year. However, cost-offset analysis revealed that additional healthcare costs resulted from the screening could not be offset by decreased disease progression. The 5-, 10-, and 15-year accumulated incremental costs of screening versus no screening were estimated to be $396,362.8, $424,907.9, and $434,903.2, respectively. As a result, the incremental cost per PACG of any stages prevented was $1464.3.

**Conclusions:**

This study represented the first attempt to address decision-maker’s budgetary concerns when adopting glaucoma screening by developing a Markov prediction model to project health outcomes and costs. Population screening combined with AI automated diagnosis for PACG in China were able to reduce disease progression risks. However, the excess costs of screening could never be offset by reduction in disease progression. Further studies examining the cost-effectiveness or cost-utility of AI-assisted glaucoma screening were needed.

**Supplementary Information:**

The online version contains supplementary material available at 10.1186/s12889-021-11097-w.

## Background

Glaucoma is the second leading cause of blindness after cataracts, affecting 13.12 million people in China [1, 2]. Among them, primary angle-closure glaucoma (PACG) is the predominant type of glaucoma, accounting for 7.14 million patients with a prevalence of 1.40% among China populations aged over 45 [2]. The number of PACG patients in China is projected to reach 7.5 million in 2020, surpassing that in India and ranking first in the world [3]. Considering the irreversibility of vision and visual field loss in glaucoma, early detection and intervention are essential for glaucoma management, which can effectively delay optic nerve damage [4, 5]. However, as the initial phase of glaucoma is usually asymptomatic, over 90% of patients in China remain unaware of their conditions until in late stage [6, 7]. Once the disease progress to late stage, the effects of medical interventions are unsatisfactory, underpinning the importance of population-based screening to identify asymptomatic glaucoma patients in communities and subsequent timely referral and treatment.

Glaucoma screening requires manually assessing optic nerve structure from digital fundus image. The process is labor-intensive and time-consuming, and the accuracy of diagnosis heavily relies on the skill and experience of ophthalmologists [8]. Currently, the problem with population-based screening for glaucoma is lack of ophthalmologists and poor ophthalmology capability at grassroot hospitals, making community screening difficult to implement [9]. The past several years has witnessed significant technology advancement of artificial intelligence (AI) in glaucoma detection [8, 10–12]. The idea is that a large amount of glaucoma specialist-labelled fundus images is used to train deep learning system (DLS) so that the algorithms can establish the association of abnormality patterns of the cup-to-disk ratio and optic disc hemorrhage specifically with glaucomatous optic neuropathy [13]. The advantages of AI automated glaucoma diagnosis are not only simple and fast, but also improved accuracy without relying on the subject judgement of experts [13, 14]. Therefore, researchers have advocated that integrating AI into community screening can overcome resource and capability deficiencies of primary care centers by providing diagnostic support to ophthalmologists [15].

However, the application and performance of AI automated diagnosis system in real-world clinical settings has been rarely reported, leaving the validity and suitability of such technology in population-based screening to be determined [16]. Moreover, when consider adoption of a new health intervention in real-world settings, policymakers not only evaluate its safety and effectiveness, but also the potential cost impact on the health system. For example, the local government of Changjiang county, Hainan province, China has decided to fund the Changjiang Medical Group of People’s Hospital of Wuhan University to design and conduct a community screening in its jurisdiction. Despite the expected increase of public health benefits from the screening program, the local authority, facing with the pressure of already skyrocketing health expenditures, was deeply concerned of the budget impact of such intervention. Whether the AI-assisted, population-based screening for glaucoma is worth financing from a budgetary perspective has not been explored to date.

This present study was aimed to address this issue by modelling the health outcomes and costs incurred by the screening program for glaucoma and comparing them with the health outcomes and costs that would have incurred if no screening had been implemented in the context of Changjiang county. Our hypothesis was that the screening program would inevitably increase health costs in the short-term as glaucoma patients of any stages would be identified and treated earlier. However, incremental costs would be offset by the long-term health benefits since early intervention would decrease risks of progression, which in turn would lead to cost-savings from less medical resource utilization. This study took solely PACG as illustrative example because: (1) PACG is the most prevalent type of glaucoma in China; (2) for simplicity; (3) most cost-effectiveness/cost-utility studies has focused primary open angle glaucoma, while PACG has rarely been investigated [17].

## Methods

### Overview of Changjiang county

Changjiang county is located in the northwest part of Hainan Province, China. It has a total land area of ​​1617 km^2^ and comprises eight townships and 179 villages. In 2017, the county has a population of 232,000, of which 47,676 (20.55%) were aged 0–14 years, 164,929 (71.09%) aged 15–64 years, and 19,395 (8.36%) aged 65 years and above. In 2017, the gross domestic product (GDP) of Changjiang county totaled 11.44 billion yuan ($1.66 billion US dollar, using an exchange rate of 6.90 yuan per dollar) [18]. The County People’s Hospital and the County Hospital of Integrated Traditional Chinese and Western Medicine are the two secondary hospitals that are responsible for the county’s majority of basic health services. The County People’s Hospital owns 315 hospital beds and 428 medical staff, including 35 deputy chief physician or chief physician (namely senior-level doctors). The County Hospital of Integrated Traditional Chinese and Western Medicine has nearly 400 beds and 402 medical staff, 48 of which were ranked as senior doctors. In 2017, there were 1,232,160 outpatient or emergency department visits, and 25,742 discharges from hospital in the county.

### Glaucoma screening programme

This project, characterized by AI-assisted diagnosis, hospital referral, and community follow-up, sets out to provide screening for PACG for residents aged 65 years and over (approximately 19,395 people) living in Changjiang county. There will be 13 screening sites (2 community health centers and 11 township health centers) throughout the county. Before screening, all communities and villages will put up posters to publicize the programme, and we also employ local media e.g. television and newspaper as propaganda. Besides, health workers from community and township health centers will contact potential subjects (i.e. aged 65 and above) via phone calls to explain the importance of glaucoma screening and invite them to participate. A project team formed by 1 ophthalmologist, 1 health technician, and 3 nurses from the two county hospitals will be sent out to screening sites to carry out the screening. The proposed diagnostic examination package of the screening was illustrated in Additional file 1: Appendix 1 in electronic supplementary materials (ESM), which includes visual acuity assessment, intraocular pressure measurement, optic disc examination based on non-mydriatic fundus camera and so on.

**Fig. 1:**
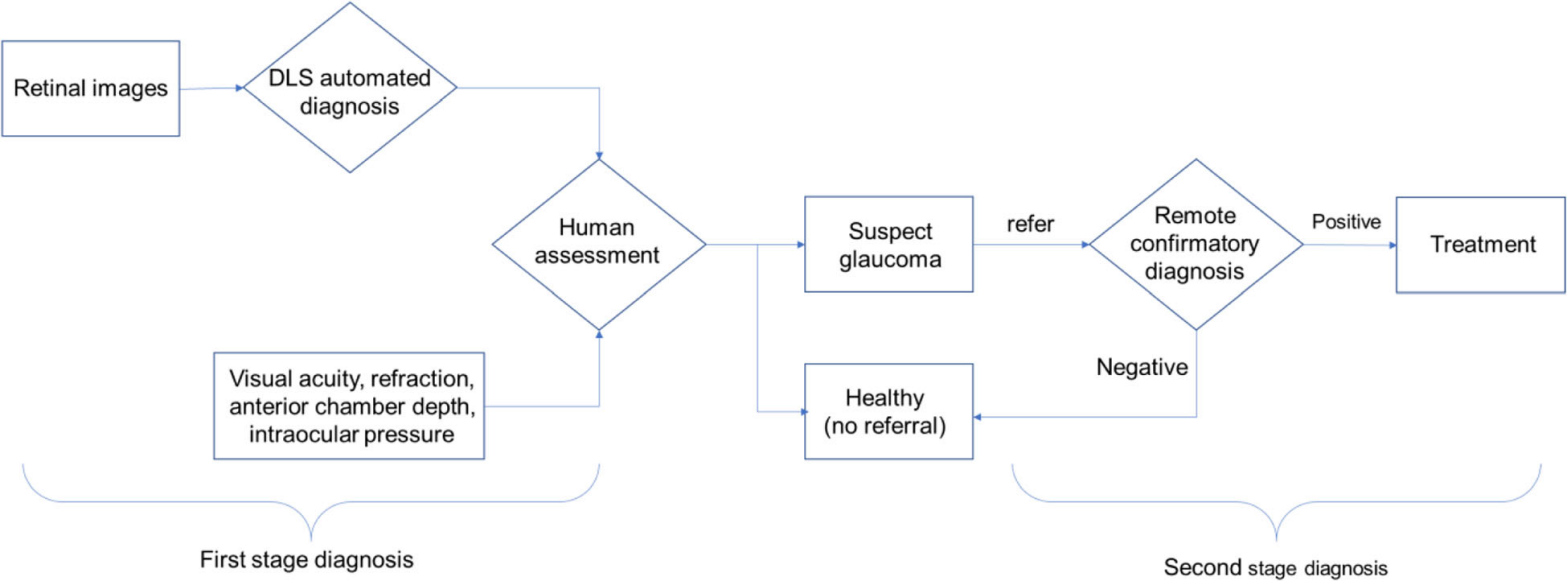
Two-stage semi**-**automated glaucoma diagnosis models using deep learning system

Figure 1 illustrated a two-stage diagnosis model adopted in the community screening. The first stage applied a hybrid diagnostic model which comprised of DLS automated grading and human assessment. A hypothetical DLS system was developed based on convoluted neural networks (CNN) approach to grade fundus images. Results from DLS automated system along with other examination data will be subsequently assessed by on-site ophthalmologists. Recommended referral will be made to participants with suspect glaucoma (i.e. referable glaucoma). Following preliminary community screening, subjects who are compliant with referral to local county hospitals will receive further examinations for confirmatory diagnosis. At this stage, glaucoma specialists from Renmin hospital of Wuhan university will remotely evaluate ophthalmic test results that are transmitted from Changjiang county. For detailed care pathways for the whole organized screening programme, please refer to Additional file 1: Appendix 2 in ESM.

### Model structure

**Fig. 2:**
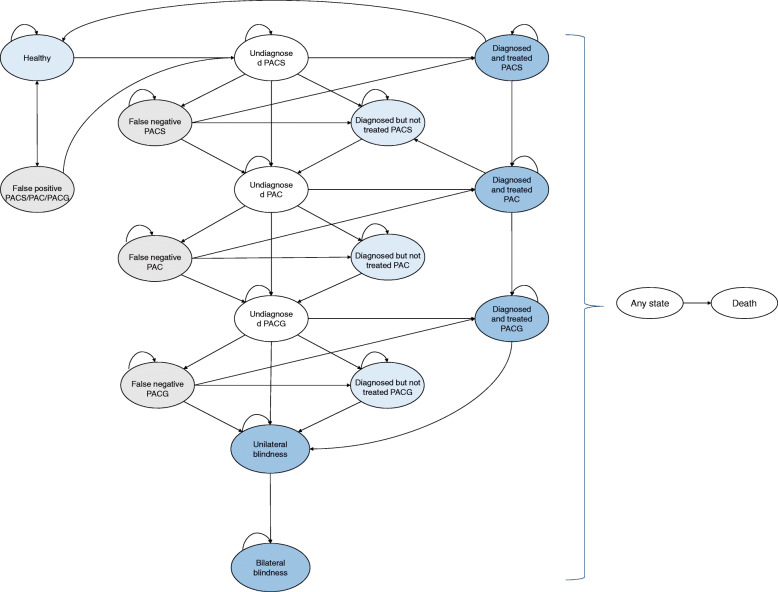
Schematic diagram of state transition in the Markov model for PACG patients under screening. *The arrows indicate the direction of transition from one state to another, and the number next to the arrow represents the corresponding transition probability*

A Markov model based on health care system’s perspective was adapted from Tang et al. and Hernández et al. to simulate the disease progression, diagnosis, treatment status of PACG patients, and the resulting resource utilization and costs over time [19, 20]. The model was simulated over a 15-year time horizon with each cycle being 1 year. A cohort of 19,395 residents aged 65 and above entered the model as healthy or affected by angle closure on the basis of their prevalences. For the screening scenario, the cost-offset model incorporated 17 states (Fig. 2). Among them, the International Society of Geographical and Epidemiologic Ophthalmology (ISGEO) glaucoma classification was employed to classify PACG into 5 stages according to severity: primary angle closure suspect (PACS), primary angle closure (PAC), primary angle closure glaucoma (PACG), PACG-related unilateral blindness, and PACG-related bilateral blindness [21]. Diseases states were further stratified by diagnosis and treatment status. Under the screening scenario, participants with screening positive results (including unreliable test results) were referred to county hospitals for confirmatory examination. True positive cases (i.e. PACS, PAC or PACG) could proceed or refuse to receive further treatment, whereas healthy participants with false positive results would never go on to unnecessary treatment. In this case, there were extra diagnostic costs but no treatment-related costs would be incurred. Patients with PACS, PAC or PACG might be mislabeled as healthy by the AI-automated system. As a result, these false negative cases would progress at the same rate as untreated patients unless they were identified in next year’s screening programme. In addition, death may occur from any states. For the opportunistic case finding scenario, a separate model with similar states was developed (Additional file 1: Appendix 3 in ESM). The discount rate for predicted healthcare costs was set at 5%.

To simplify the model, the model followed several assumptions: (1) PACS, PAC or PACG patients who were not compliant with recommended treatment at county hospitals would remain in the diagnosed but not treated state until disease progression; (2) Patients could only be treated once at each stage; (3) A large-scale randomized clinical trials with 6-year follow up has revealed that patients with PACS or PAC who had underwent treatment could be reversed back to earlier stages [22]. Hence treated PACS was allowed to transition back to healthy state. Similarly, treated PAC could return to PACS with the same disease progression risks as treatment-naïve PACS; (4) Blindness was assumed to occur in a linear way. That is, it would first be unilateral and then become bilateral; (5) We only considered the diagnostic accuracy of the DLS automated system in this study; (6) It was assumed that all previously treated patients would be closely follow-up. Once progressed to next stages of disease, they received corresponding treatments immediately; (7) All patients with either unilateral or bilateral blindness would receive clinical treatment.

### Epidemiology

Tang et al., through meta-analysis, reported that prevalence rates of PACS, PAC, and PACG in the overall population in rural areas of China were 8.618, 1.797, and 0.592%, respectively [19]. Considering that Changjiang County had a population of 19,395 people over 65 years of age, it was estimated that there were 1671, 349, and 115 patients with PACS, PAC, and PACG among the elderly population at baseline, respectively.

### Transition probability

Transition probabilities were differed by treatment status since clinical interventions were supposed to reduce disease progression risks (Table 1). We firstly derived progression risks from randomized clinical trials or cohort studies conducted in China. Transition probabilities of clinical events for which Chinese-specific data were not available were complemented by studies done in other Asian populations that share similar ethnic profiles. For progression risks which were measured over time periods greater than 1 year, the model firstly converted these multi-year probabilities to rates via the formula rate = − Ln (1 - t-year probability) / t years, and then calculate the 1-year probabilities through 1-year probability = *e*^(−*rate* ∗ 1)^, where t > 1. This approach was based on exponential survival distributions which assumed that for each event, event hazards were constant over time [28].

**Table 1:** Disease progression risks between stages, by treatment status

Between states transition	Untreated		Treated	
	Probability	Source	Probability	Source
**From Health**				
to PACS	1.83%	Wang et al .[23]	–	–
**From PACS**				
to PAC	4.85%	Thomas et al .[24]	1.14%	Yip et al .[22]
to health	–	–	18.69%	Yip et al .[22]
**From PAC**				
to PACS	–	–	1.01%	Yip et al .[22]
to PACG	6.51%	Thomas et al .[25]	2.06%	Yip et al .[22]
**From PACG**				
to unilateral blindness	2.98%	Tang et al .[19]	0.49%	Quek et al .[26]
**From unilateral blindness**^a^				
to bilateral blindness	–	–	2.60%	Rossetti et al .[27]

Note: ^a^: assumed all unilateral blindness would receive treatment

### Compliance

It was assumed that 60% of eligible residents aged over 65 in Changjiang county would participate in the screening, and this assumption was tested in sensitivity analysis. A previous population-based screening study has reported that compliance with hospital referral for confirmatory diagnosis was 48.9% [29]. The compliance of early-stage patients (PACS and PAC) receiving clinical interventions differed between screening and no screening scenarios. It was worth mentioning that within each scenario, treatment compliance of PACS and PAC were assumed to be the same (Table 2).

**Table 2:** Compliance with participation, hospital referral and treatment

	Value	Source
Community screening participation rate	60.0%	assumed
Compliance with hospital referral	48.9%	Zhang et al. [29]
Compliance with treatment among PACS and PAC		
Screening scenario	32.1%	Thomas et al. [25]
No screening scenario	20.0%	Assumed
Compliance with treatment among PACG		
Screening scenario	80.0%	Tang et al. [19]
No screening scenario	42.9%	Liang et al. [30]
Compliance with treatment among blindness	100.0%	Assumed

### Diagnostic accuracy of AI automated diagnosis system

We performed a meta-analysis to derive the sensitivity and specificity of DLS automatic diagnosis system in identifying referable glaucoma (Table 3). Eligibility was restricted to studies that met the following criteria: (1) aimed to evaluate the performance of DLS in detecting referable glaucoma, with the same diagnostic criteria applied; (2) the DLS model were developed on the basis of CNN approach from digital fundus images; (3) conducted in China so that both training and validating images for machine learning fully reflected the fundus characteristics of Chinese patients. For more details about search strategy and results of pooled analysis, please refer to Additional file1: Appendix 4 in ESM.

**Table 3:** Sensitivity and specificity of the DLS automatic diagnosis for glaucoma

Parameter	Probability	Source
Sensitivity	95.9%	Internal meta-analysis
Specificity	96.1%	Internal meta-analysis

### Costs

Two cost components were considered in our study: equipment acquisition and clinical treatment (Table 4). Units and unit costs of screening equipment were listed in Additional file 1: Appendix 5. It was assumed that all screening equipment had a useful lifespan of 5 years, and costs of equipment were annualized using the straight-line depreciation method. Patients confirmed as PACS, PAC or PACG at two county hospitals were recommended to received clinical interventions according to the China guidelines [31]. Specifically, the first-line treatment was laser peripheral iridotomy (LPI) for PACS and PAC patients. Among them, 9.8% of PACS whose intraocular pressure would rise again and thus require further medications, whereas 41.3% of PAC would fail the surgery and also need drug therapy [32, 33]. For PACG patients, trabeculectomy was provided. After surgery, the proportion of PACG who still required long-term topical medications was assumed to be the same as that of PAC (41.3%). All surgeries were performed during hospitalization. Besides the surgery itself, hospitalization costs also included examination, medication, medical consumables, nursing, hospital bed-day etc. Long-term medications were prescribed in the outpatient setting. Note that costs of long-term medications were converted on an annual basis.

**Table 4:** Annualized equipment and treatment costs

Cost items	Annual Costs ($)	Source
**Equipment**		
Equipment costs	74,654.9	Internal data
**Treatment**		
Hospital examination	15.9	Tang et al. [19]
PACS or PAC		
Outpatient medications	1.8	Tang et al. [19]
Inpatient surgery	105.0	Tang et al. [19]
PACG		
Outpatient medications	10.6	Tang et al. [19]
Inpatient surgery	565.6	Electronic medical records
Unilateral blindness		
Outpatient medications	347.9	Tang et al. [19]
Inpatient surgery	976.6	Electronic medical records
Bilateral blindness		
Outpatient medications	347.9	Tang et al. [19]
Inpatient surgery	1233.5	Electronic medical records

Note: Electronic medical records were obtained from the two county hospitals in the County People’s Hospital and the County Hospital of Integrated Traditional Chinese and Western Medicine

### One-way sensitivity analysis

All parameters in the Markov model were subject to one-way sensitivity analysis. Parameters were varied between their 95% confidence interval (CI) whenever available. For those parameters for which 95% CI were not reported from source studies, they were to be increased and decrease by 20% of their original value.

## Results

### Predicted long-term health outcomes of glaucoma patients

**Fig. 3:**
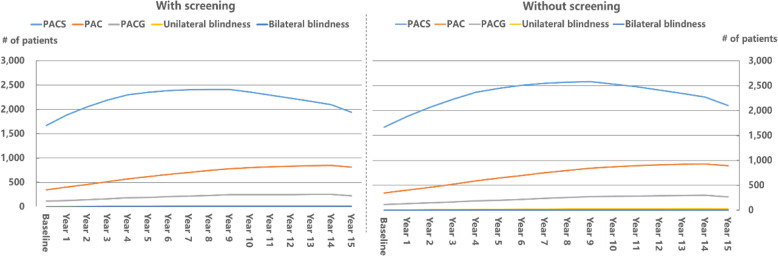
Plot of distribution of glaucoma patients over a 15-year horizon under two scenarios: with screening versus without screening

Figure 3 and Table 5 gave the number and distribution of PACG patients over the next 15 years predicted by the Markov model. At baseline, there were 1671, 349, and 115 patients with PACS, PAC, and PACG, respectively. The number of patients with PACS over time was featured with a concave downward shape, meaning PACS patients rose with a decreasing growth rate in the first several years, but ultimately declined. In year 15, the number of PACS under the screening were estimated to be 1940, which was 7.6% less than that under no screening (2104). Unlike PACS, the number of patients with PAC, PACG, unilateral and bilateral blindness were steadily increased except for the 15th year, regardless of the presence or absence of the screening. However, the growth rates of the patient numbers were notably lower in the screening scenario than those without screening. In year 1, both scenarios had 402 PAC, 129 PACG, 3 unilateral blindness, 0 bilateral blindness. Fourteen years later, the projected numbers of patients with PAC, PACG, unilateral and bilateral blindness under the screening increased to 816, 224, 16 and 2. By contrast, the corresponding numbers under no screening were 895, 269, 24, and 3, respectively. In other words, the screening programme would reduce the number of PAC by 8.8%, PACG by 16.7%, and blindness (unilateral and bilateral blindness combined) by 33.3% compared with the no screening scenario over a 15-year period. Overall, the screening programme was predicted to decrease 297 (− 9.0%) disease cases of any stages over a 15-year horizon.

**Table 5:** Stage distribution of glaucoma patients under the screening scenario versus no screening scenario, by year

Year	Screening					No screening				
	PACS	PAC	PACG	Unilateral blindness	Bilateral blindness	PACS	PAC	PACG	Unilateral blindness	Bilateral blindness
Baseline	1671	349	115	0	0	1671	349	115	0	0
Year 1	1883	402	129	3	0	1883	402	129	3	0
Year 2	2052	457	145	6	0	2068	461	146	7	0
Year 3	2188	513	163	9	0	2230	524	166	10	0
Year 4	2300	570	182	11	0	2370	589	188	13	0
Year 5	2352	617	193	13	1	2449	645	202	15	1
Year 6	2386	662	205	15	1	2508	699	219	18	1
Year 7	2405	705	218	16	1	2547	751	236	20	1
Year 8	2412	745	232	17	1	2571	800	255	22	2
Year 9	2409	782	246	18	2	2581	845	274	25	2
Year 10	2356	803	247	18	2	2535	872	280	26	2
Year 11	2297	820	248	18	2	2479	894	285	26	3
Year 12	2233	833	250	18	2	2416	910	292	27	3
Year 13	2166	842	253	18	2	2346	922	297	28	3
Year 14	2097	848	255	18	2	2273	930	303	28	3
Year 15	1940	816	224	16	2	2104	895	269	24	3

### Predicted long-term healthcare costs and cost-offset analysis

**Fig. 4:**
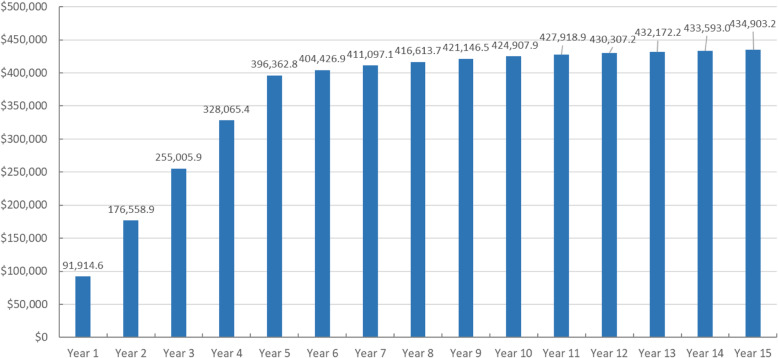
Cumulative differences of total healthcare costs between the two scenarios in Changjiang County (US dollar)

Table 6 showed annual healthcare costs and Fig. 4 displayed the cumulative differences of healthcare costs between the two scenarios. Healthcare costs were estimated to surge dramatically to $107,761.4 dollar in the first year under the screening, compared with $15,846.8 dollar without screening. The increase in costs with screening was mostly driven by the annualized capital costs for equipment. The annual healthcare costs with screening were projected to decline over time. Specifically, the annual cost dropped significantly from $84,430.5 in the 5th year to $24,544.0 in the 6th year since the depreciation of capital equipment has been completed. Medical expenses under no screening showed an upward from the second year ($14,759.8) until reaching the peak ($17,900.9) in the 9th year. The model pointed out that costs with screening could never be offset by the reduction in cases in any year compared with the no screening scenario. As a result, relative to the no screening scenario, incremental costs due to the screening programme accumulated to $396,362.8, $424,907.9, and $434,903.2 over a 5-, 10- and 15-year horizon, respectively.

**Table 6:** Results of cost-offset analysis, by year (US dollar)

Year	Screening							No screening						Difference
	Annualized capital cost	PACS	PAC	PACG	Unilateral blindness	Bilateral blindness	Total	PACS	PAC	PACG	Unilateral blindness	Bilateral blindness	Total	
1	71,098.96	15,142.2	3174.4	14,029.6	4316.2	0.0	107,761.4	2170.3	456.3	8903.9	4316.2	0.0	15,846.8	91,914.6
2	67,713.30	12,214.5	2821.8	11,688.8	4838.0	127.7	99,404.1	2198.3	501.0	6812.4	5120.4	127.7	14,759.8	84,644.3
3	64,488.85	10,173.3	2595.6	10,354.9	5353.1	250.8	93,216.5	2191.2	543.4	5802.5	5973.4	259.1	14,769.6	78,446.9
4	61,417.96	8706.0	2447.9	9658.9	5861.6	371.2	88,463.6	2157.2	581.8	5407.8	6860.7	396.6	15,404.1	73,059.5
5	58,493.29	7613.0	2346.0	9345.0	6151.7	481.5	84,430.5	2103.0	614.9	5347.2	7535.9	532.1	16,133.1	68,297.4
6	0.00	6644.6	2233.1	8841.1	6260.8	564.3	24,544.0	1999.8	632.4	5211.1	7991.5	645.1	16,479.8	8064.1
7	0.00	5874.5	2137.0	8611.6	6363.4	634.9	23,621.3	1889.1	643.3	5244.7	8424.0	750.0	16,951.1	6670.2
8	0.00	5241.8	2049.1	8517.8	6455.1	695.7	22,959.5	1774.8	648.0	5343.8	8828.4	847.8	17,443.0	5516.5
9	0.00	4707.8	1964.6	8480.5	6532.6	748.3	22,433.8	1659.9	647.1	5454.5	9200.1	939.4	17,900.9	4532.9
10	0.00	4246.0	1879.6	8435.6	6330.4	768.8	21,660.5	1546.2	640.7	5537.6	9180.0	994.6	17,899.1	3761.3
11	0.00	3773.3	1765.4	8019.8	6007.6	756.8	20,322.9	1410.6	620.0	5335.7	8938.5	1007.1	17,311.9	3011.0
12	0.00	3360.7	1654.2	7701.1	5719.9	739.2	19,175.0	1283.3	596.2	5192.3	8704.7	1010.1	16,786.7	2388.3
13	0.00	2997.6	1546.1	7418.8	5457.0	718.1	18,137.5	1164.6	570.0	5059.7	8472.0	1006.2	16,272.6	1865.0
14	0.00	2676.5	1441.4	7144.3	5211.4	695.1	17,168.8	1054.7	542.2	4918.6	8235.4	997.0	15,748.0	1420.8
15	0.00	2388.6	1337.1	6827.4	4488.1	611.1	15,652.3	952.6	511.9	4737.9	7238.8	900.8	14,342.1	1310.2

### One-way sensitivity analysis results

**Fig. 5:**
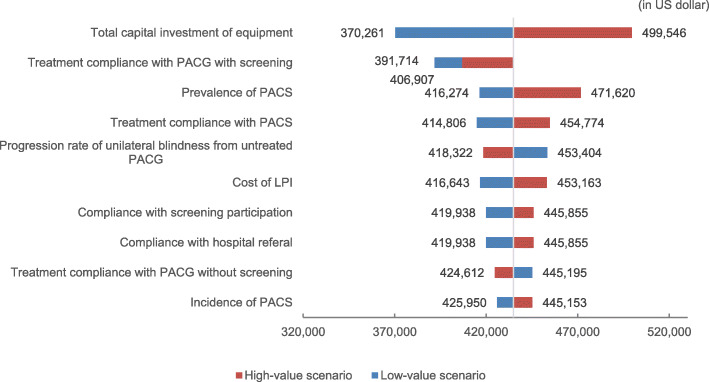
Tornado plot of 15-year accumulated incremental costs of screening versus no screening (only presented the top ten parameters that had the largest impact)

One-way sensitivity analysis revealed that total capital investment of screening equipment, treatment compliance with PACG under screening, and prevalence of PACS under screening were the top 3 parameters that impacted most significantly the15-year accumulated incremental costs (Fig. 5). By adjusting parameters to their extremes, the 15-year accumulated incremental cost of screening relative to no screening varied within 15% around the base-case value ($434,903.2).

## Discussion

To our best knowledge, this was the first study evaluating community glaucoma screening from a budgetary standpoint. This study set out to address decision-makers’ budget concerns when adopting a glaucoma screening, by developing a Markov prediction model with PACG as illustrative example in which medical care costs under the screening were projected and compared against what they would have been without screening. Our study found that among 19,395 residents aged 65 and above in Changjiang county, the AI-assisted community screening for PACG was able to reduce patients with PACS by 163 (7.7%), PAC by 79 (8.8%), PACG by 45 (16.7%), and prevent 10 patients (33.3%) from any visual blindness. However, additional healthcare costs resulted from the community screening could not be offset by decreased disease progression. The 5-, 10-, and 15-year accumulated incremental costs of screening, compared with the no screening scenario, were estimated to be $396,362.8, $424,907.9, and $434,903.2, respectively. Combing the disease case numbers and incremental costs, our results indicated that the incremental cost per disease case of any stages prevented was $1464.3 over a 15-year horizon.

We also found that although the programme could not lead to cost-saving, there was a consistent downward trend in medical care costs with screening. This related to the fact that with screening, patients at earlier stages (PACS or PAC) would be identifier and treated in a timely manner so that disease progression would be prevented or delayed. Considering the fact that patients seeking medical treatment outside of the Changjiang county were not reflected in our model, we believed that the declining rate of cost might be more pronounced in real-world setting. This was because many patients screened positive in community would be directly referred through green channels to county hospitals for diagnosis and treatment. As a result, the number of patients seeking treatment in tertiary hospitals outside of the county which were generally associated with expensive healthcare services would drop.

The economical profile of glaucoma screening was still controversial in the academic community. Most studies focused on the cost-effectiveness of glaucoma screening. Previous studies by British researchers found that the incremental cost-effectiveness ratio (ICER) of population screening exceeded £30,000 per additional quality-adjusted life year (QALY) gained, making it less cost-effective than no screening [20, 34, 35]. A recent study in the United States favored economically glaucoma screening based on routine physical checkup, with an ICER of $46,000 per QALY gained [36]. In developing countries, an India study suggested that population-based glaucoma screening might be a cost-effective alternative to no screening [17]. Similar results were also observed in China [19]. On top of the cost-effectiveness aspect, our study added to evidence by demonstrating that an glaucoma screening with AI automated system could not reduce health care expenses induced by glaucoma disability. The 15-year accumulated incremental cost amounted to $434,903.2, which translated into $1464.3 per PACG of any stages prevented. Whether such excess costs were bearable depended on the local government’s financial resources. One possible explanation for the excess costs was due to the low rates of natural progression of PACS, PAC, and PACG, making the benefits of early diagnosis and treatment less pronounced compared with the standard of care. Therefore, it might require a longer time horizon for the cost-savings of screening to be realized. Another explanation might be the substantial upfront capital investment. As our results pointed out, capital costs for equipment represented the most significant driver for cumulative healthcare costs, indicating necessity for optimizing capital equipment purchasing.

China is currently undergoing population aging and transition from infectious diseases to non-infectious chronic diseases. The health system is facing a dilemma of limited health resources and increasing demand for high quality health services. Based on this background, there is an urgent need to create an efficient disease management model that shifts the focus from hospital care to public health services e.g. community screening for chronic diseases. Unlike clinical interventions such as drugs and medical devices that directly affect the pathogenies, the effect of public health is indirect since it works by regulating risk factors to control onset and progression of diseases. Therefore, benefits of public health are not as immediate as clinical treatments. Given this long-term nature, evaluation of public health interventions requires longer observation periods and significant investment in personnel and financial resources. This may explain why there is lack of evidence on the impact of population screening [37]. In this case, Markov prediction models represent a good alternative, which combine mathematical algorithms with clinical trial and epidemiological evidence to project the impact of health interventions on the health system thereby supporting health decision-making. To the best of our knowledge, our study was the first attempt to utilize modelling technique to predict the budgetary impact of AI-assisted glaucoma screening on healthcare costs. Nevertheless, there were some inevitable limitations in this study. Firstly, as a modelling study, the predictive accuracy relied on parameters used in the Markov model. There were potential risks that parameters obtained from existing studies conducted in other regions could not be extrapolated to Changjiang county. To test the robustness of our model, we employed one-way sensitivity analysis to quantify the impact of all parameters on prediction results. Secondly, there was a lack of concrete evidence on the efficacy of treatment (e.g. LPI) for PACG due to absence of high quality randomized clinical trials. We recognized that this might be a challenge and thus we strived to base our model on the best available evidence. Additionally, the fact that all evidence sources for the treatment effectiveness included in this study have also been cited in other cost-effectiveness analysis has augmented their validity [19]. Thirdly, we did not consider the impact of newly emerging health technologies and drugs. Fourthly, although various image-based AI automated diagnosis systems for ophthalmic disease were greatly advanced in recent years, their diagnostic performance have not been examined with a rigorous experimental study design.

## Supplementary Information

**Additional file 1: Appendix 1.** Protocol of community screening tests. **Appendix 2.** Flowchart of AI-assisted population-based glaucoma screening and hospital referral versus opportunistic case finding in Changjiang County. **Appendix 3.** Schematic diagram of health state transition in the Markov model for glaucoma patients without screening. **Appendix 4.** Pooled analysis of performance of AI automated diagnosis system in detecting referable glaucoma. **Appendix 4.1.** Data extraction from included studies. **Appendix 4.2.** Pooled analysis results. **Appendix 4.3.** Forest plot of sensitivity and specificity estimates from 4 studies. **Appendix 5.** Capital costs of equipment spent in the screening programme.

## Data Availability

All data analyzed or generated during this study were obtained from published articles or from internal data from the Changjiang screening programme. All data are freely available on reasonable request by contacting the corresponding author.
