# Supplementary material for: Health care cost and benefits of artificial intelligence-assisted population-based glaucoma screening for the elderly in remote areas of China: a cost-offset analysis
Source: BMC Public Health. 2021 Jun 4;21:1065. doi: 10.1186/s12889-021-11097-w (PMC8178835; doi:10.1186/s12889-021-11097-w)
Supplement: Supplementary file 1 — Additional file 1: Appendix 1. Protocol of community screening tests. Appendix 2. Flowchart of AI-assisted population-based glaucoma screening and hospital referral versus opportunistic case finding in Changjiang County. Appendix 3. Schematic diagram of health state transition in the Markov model for glaucoma patients without screening. Appendix 4. Pooled analysis of performance of AI automated diagnosis system in detecting referable glaucoma. Appendix 4.1. Data extraction from included studies. Appendix 4.2. Pooled analysis results. Appendix 4.3. Forest plot of sensitivity and specificity estimates from 4 studies. Appendix 5. Capital costs of equipment spent in the screening programme. [file 12889_2021_11097_MOESM1_ESM.docx]

**Health care cost and benefits of artificial intelligence-assisted population-based glaucoma screening for the elderly in remote areas of China: A cost-offset analysis**

Xuan Xiao^1^, Long Xue^2^, Lin Ye^3^, Hongzheng Li^2^, Yunzhen He^*,2^

^*^Corresponding author: Yunzhen He, [yogurtfdu@gmail.com](mailto:yogurtfdu@gmail.com)

^1^Eye Center, Renmin Hospital of Wuhan University, 430060 (Xuan Xiao)

^2^School of Public Health, Fudan University, 200131 (Yunzhen He, Long Xue, Hongzheng Li);

^3^Department of Eye Plastic and Lacrimal Disease, Shenzhen Eye Hospital of Jinan University, Shenzhen, China, 518040 (Lin Ye)

**
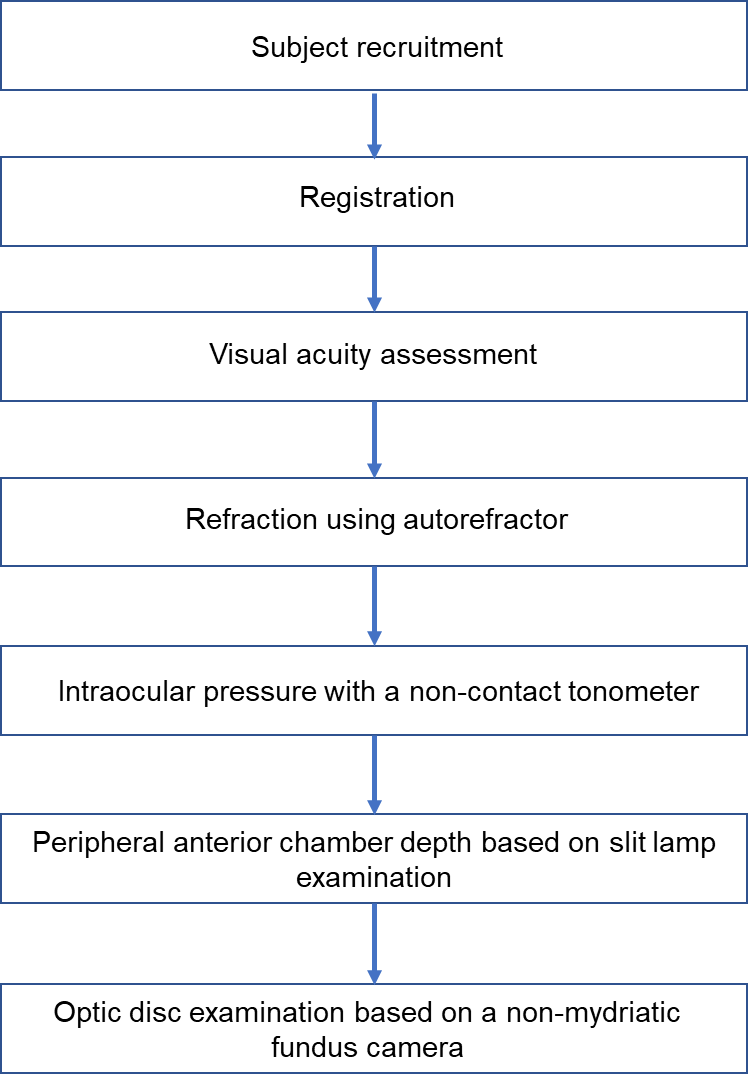
Appendix 1. Protocol of community screening tests**

**Appendix 2. Flowchart of AI-assisted population-based glaucoma screening and hospital referral versus opportunistic case finding in Changjiang County**


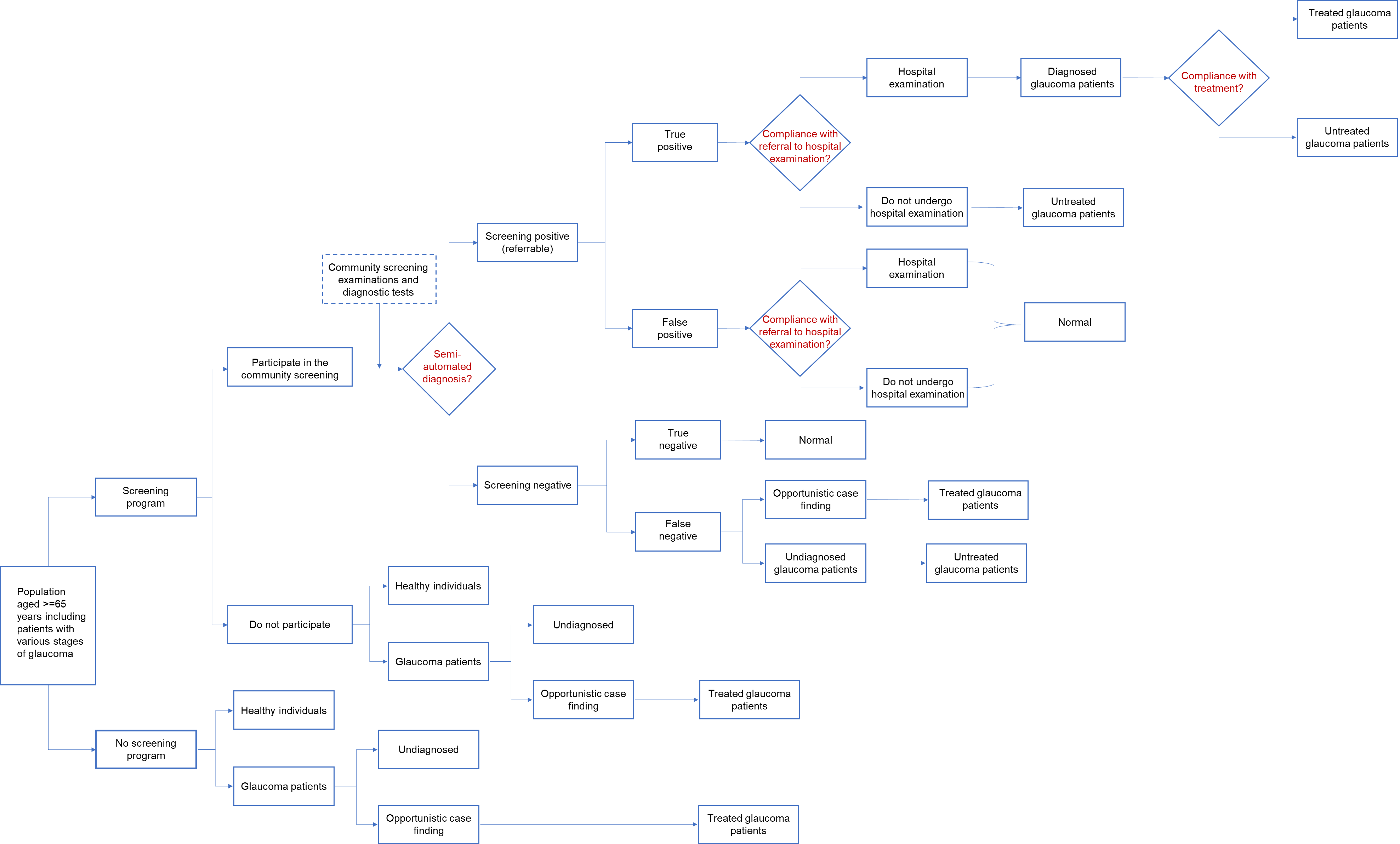


**Appendix 3. Schematic diagram of health state transition in the Markov model for glaucoma patients without screening**

**
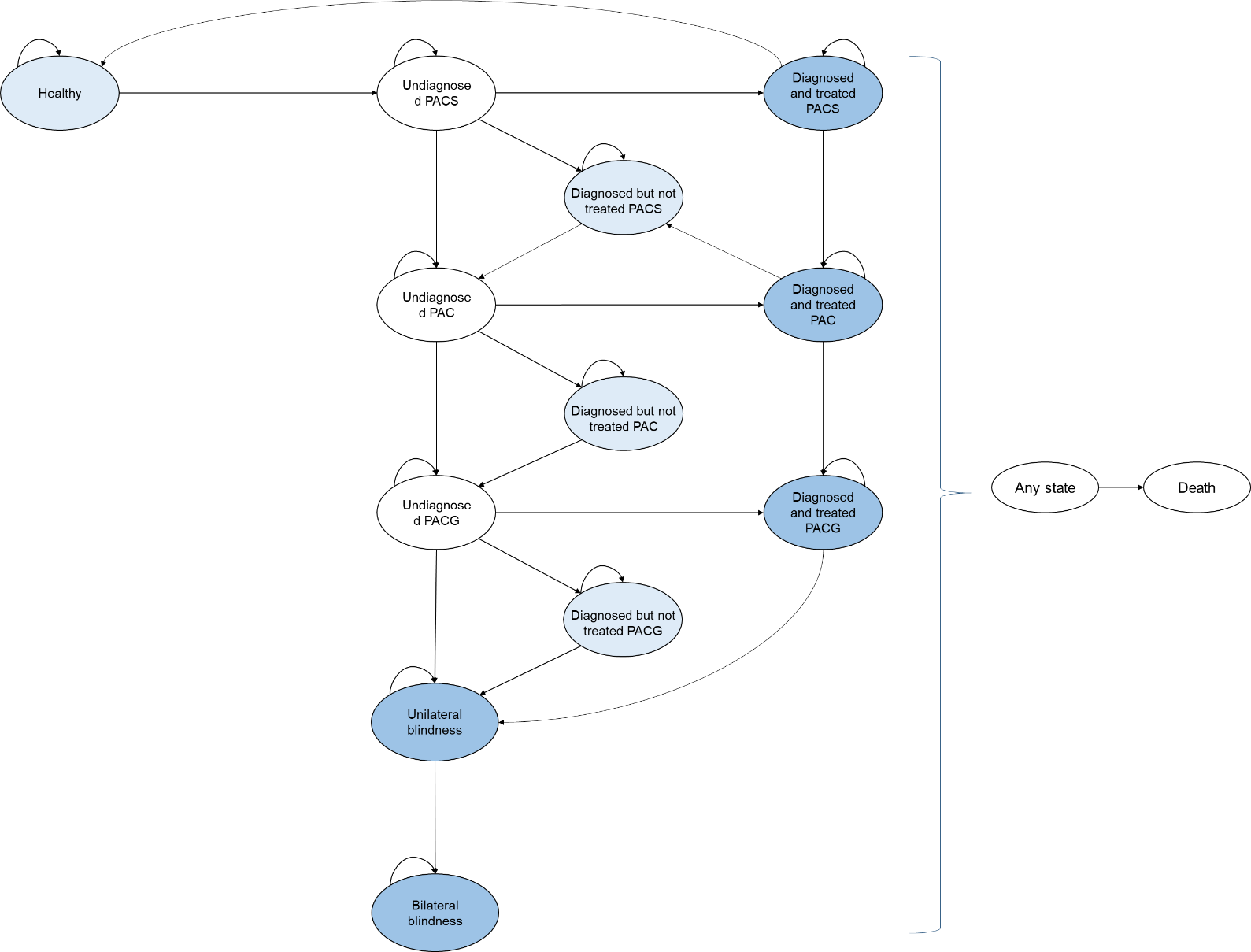
**

**Appendix 4. Pooled analysis of performance of AI automated diagnosis system in detecting referable glaucoma**

We conducted a systemic search in PubMed to identify relevant articles, with the following search strategy: ("glaucoma"[tiab] OR "glaucomatous optic neuropathy"[tiab] OR "GON"[tiab] OR "glaucomatous disease"[tiab]) AND ("artificial intelligence"[tiab] OR "deep learning"[tiab] OR "AI"[tiab] OR "machine learning"[tiab]) AND ("fundus"[tiab] OR "retina"[tiab]) AND ("china"[tiab] OR "chinese"[tiab]).

4 studies were indexed from the database, all of which met our eligibility criteria and thus were included in our pooled analysis. Data on true positive, true negative, false positive and false negative cases were extracted from these 4 studies and subsequently synthesized with a random effect model in Stata 14.0 version, using MIDAS, a user-written command for meta-analysis of diagnostic test performance.

| **Appendix 4.1 Data extraction from included studies** | | | | | |
| --- | --- | --- | --- | --- | --- |
| **Studies** | **Year** | **True positive** | **False positive** | **False negative** | **True negative** |
| Liu et al^1^ | 2019 | 4892 | 539 | 193 | 22888 |
| Li et al^2^ | 2018 | 1880 | 483 | 87 | 5550 |
| Li et al^3^ | 2020 | 1873 | 108 | 84 | 1416 |
| Li et al^4^ | 2020 | 115 | 11 | 3 | 654 |

| **Appendix 4.2 Pooled analysis results** | | |
| --- | --- | --- |
| **Parameter** | **Pooled estimates** | **95% CI** |
| Sensitivity | 0.96 | 0.95 – 0.96 |
| Specificity | 0.96 | 0.92 – 0.98 |
| Positive likelihood ratio | 24.4 | 12.4 – 47.9 |
| Negative likelihood ratio | 0.04 | 0.04 – 0.05 |
| Diagnostic odds ratio | 581 | 263 - 1287 |

**Appendix 4.3 Forest plot of sensitivity and specificity estimates from 4 studies**

**Appendix 5 Capital costs of equipment spent in the screening programme**

| Equipment | Units | Unit costs ($) | Usage life (years) | Annualized cost ($/year) |
| --- | --- | --- | --- | --- |
| Topcon 3D Optical Coherence Tomography (OCT) 2000 (Topcon, Tokyo, Japan) | 1 | 173,951 | 5 | 34,790 |
| SL-3G Slit Lamp (Topcon, Tokyo, Japan) | 1 | 17,395 | 5 | 3,479 |
| TRC-50DX Fundus Camera (Topcon, Tokyo, Japan) | 1 | 130,435 | 5 | 26,087 |
| Non-contact computerized Tonometer CT-800 (Topcon, Tokyo, Japan) | 1 | 27,542 | 5 | 5,508 |
| Auto Kerato-Refractometer KR-800 (Topcon, Tokyo, Japan) | 1 | 21,739 | 5 | 4,349 |
| Laptop | 3 | 435 | 5 | 261 |

**Reference**

[1] Liu H, Li L, Wormstone IM, Qiao C, Zhang C, Liu P, Li S, Wang H, Mou D, Pang R, Yang D. Development and validation of a deep learning system to detect glaucomatous optic neuropathy using fundus photographs. Jama Ophthalmology. 2019 Dec 1;137(12):1353-60.

[2] Li Z, He Y, Keel S, Meng W, Chang RT, He M. Efficacy of a deep learning system for detecting glaucomatous optic neuropathy based on color fundus photographs. Ophthalmology. 2018 Aug 1;125(8):1199-206.

[3] Li F, Yan L, Wang Y, Shi J, Chen H, Zhang X, Jiang M, Wu Z, Zhou K. Deep learning-based automated detection of glaucomatous optic neuropathy on color fundus photographs. Graefe's Archive for Clinical and Experimental Ophthalmology. 2020 Jan 27:1-7.

[4] Li Z, Guo C, Lin D, Nie D, Zhu Y, Chen C, Zhao L, Wang J, Zhang X, Dongye M, Wang D. Deep learning for automated glaucomatous optic neuropathy detection from ultra-widefield fundus images. British Journal of Ophthalmology. 2020 Sep 16.
